# Modelling evolution of a large, glacier-fed lake in the Western Indian Himalaya

**DOI:** 10.1038/s41598-023-28144-8

**Published:** 2023-02-01

**Authors:** Prateek Gantayat, RAAJ Ramsankaran

**Affiliations:** 1 Hydro-Remote Sensing Applications (H-RSA) Group, Department of Civil Engineering, Indian Institute of Technology, Mumbai, 400076 India; 2 Present Address: Independent Reasercher, Odisha, India

**Keywords:** Climate sciences, Cryospheric science

## Abstract

In this study, we simulated the evolution of a large glacier-fed lake called the Gepan Gath lake located in Western Himalayas by numerically modelling the evolution of the Gepan Gath glacier that feeds the lake. Due to the extremely large volume and steep lake sidewalls, the lake has been classified as ‘critical’ or prone to hazards such as lake outburst floods in the future, by various scientific investigations. This modelling was carried out by a 1D model that is based on the principle of mass conservation. The 1D model was forced with the glacier surface mass balance (SMB). Due to non-availability of published in-situ estimates, the SMB was estimated using an energy balance-based model on station derived and reanalysis derived meteorological data. Modelled glacier length fluctuations for over 134 years matched reasonably well with that of observed within the RMSE error ~ 320 m. In addition to that, between 2004 and 2019, the modelled and observed lake lengths were in agreement with each other with the RMSE ~ 110 m. Modelled glacier lake lengths also match well with published, satellite imagery derived lengths within 15% uncertainty. The uncertainty in future lake length fluctuations is within 100–200 m. Our ultimate aim is to show that numerical ice-flow modelling can be an asset in modelling glacier-fed lake evolution even in the case of highly data-sparse regions of the IHR.

## Introduction

Proglacial lakes are water masses that form at glacier or ice sheet termini. Such lakes are formed when (a) glacier melt water is dammed by terminal moraines or, (b) glacier melt water is accumulated in the bed depressions that are exposed during glacier retreat. These lakes are continuously fed by glacier melt water. Himalaya is home to a large number of such lakes. Misra and Kumar^1^ identified 958 lakes located in the western Indian Himalaya out of which ~ 345 lakes are classified as glacier-fed lakes. Various studies have shown that recent glacier retreat has led to an increase in the number of glacier-fed lakes^2–5^. Investigations conducted by^6^ and^5^ showed that the majority of the glacial lakes found in the Third Pole (glacial regions of in and around the Tibetan plateau), is located in the Himalaya^6^ also estimated an increase of 14% in the glacial-lake area between 1990 and 2015 in Himalaya. In another independent study^3^, showed that in total over two thousand lakes in the Himalaya grew by ~ 123 km^2^ between 1990 and 1999 and they grew further by ~ 32 km^2^ between 2015 and 2018. A recent study conducted by^7^ reported that out of the 251 lakes in the Indian Himalayan Region (IHR), 12 are in critical state and 91 are in potentially critical state. The latest investigation by^8^ reported 73 glacial lakes in the Indian Himalaya are at high or very high risk of dam failure. Therefore, knowledge about the evolution of such lakes is essential. We believe that numerical flowline modelling of glacier retreat could be a convenient and reliable tool in this direction given that similar studies have been carried out in the past with respect to tidewater Icelandic glaciers^9^. However, such studies were not based in the IHR and were also focused on glaciers with a very simple geometry. Though numerical ice-flow based studies have been conducted in the Himalaya earlier^10–13^, the studies were mainly focused towards simulating glacier retreat as none of the test cases had a terminal lake. Simulation of past and future glacier evolution can be helpful in understanding how these glacier-fed lakes evolve. Such studies will also help understand the role of glacier-fed terminal lakes in influencing glacier retreat. Recently^14^, delineated the potential location and the plausible maximum extent of the existing and future lakes located in the Chandra Basin in Western Indian Himalaya but did not give the time of formation and the subsequent rate of growth of these lakes.

Therefore, in this study for the first time we have simulated the evolution of a large glacier-fed lake in the IHR and that too for a geometrically complex glacier using freely available satellite, reanalysis data and a numerical ice flow model. We believe that this study will help us understand the following research question:

What is the role of a terminally located, glacier-fed lakes in accelerating glacier retreat and what is the maximum extent to which these lakes can grow in the future?

In an attempt to answer the above question, we model the evolution of a large glacier-fed lake called the Gepan Gath lake using a numerical ice-flow model. The Gepan Gath lake is one of the biggest glacial lakes in the Chandra basin which is located in the Western Indian Himalaya at the terminus of the Gepan Gath glacier^15^. Studies have shown that over the period spanning 1971–2014, the lake area and lake volume has increased from 0.1 to 0.6 km^2^ and 1.9 to 23.6 million m^3^ respectively^15^. The lake is fed by the melt from the Gepan Gath glacier that has an areal extent of ~ 14 km^2^ and length ~ 6 km at present. Another major reason for choosing this lake is that, according to the recently conducted study by^16^ the Gepan Gath lake is the largest lake in the Western Indian Himalaya. The same study also predicted that as the lake expands due to future glacier retreat, the potential of dam breaching for the Gepan Gath lake increases. This increase in the dam breaching potential proportionally increases the susceptibility of many villages such as Sisu (located 10 km downstream) to floods of catastrophic proportions.

The major objectives of this study are as follows:

1. Estimate past evolution of Gepan Gath glacier using a coupled mass balance, 1-D numerical ice flow model.
2. Using the past glacier length changes, calculate the past evolution of Gepan Gath lake.
3. Estimate the future evolution of the Gepan Gath lake under different climate (RCP) scenarios.

The article is divided into six sections. Following this introduction, in the second section, we briefly talk about the study area i.e. the Gepan Gath glacier and the Gepan Gath lake. In the third section we describe the methods that were used for estimating surface mass balance and lake evolution simulations. Next, in Section “Results”, we discuss the results where it is shown how the glacier and its glacier-fed lake have evolved over the past 134 years. In the next section (Sect. 5), we discuss the results of the simulations of the future evolution of the lake under different climatic scenarios and also examine the influence of lake expansion/retreat on glacier mass loss. In section “Uncertainty analyses”, we analyse the uncertainty associated with the future lake length fluctuations. Finally (in Section “Uncertainty analyses” and “Discussion”), we highlight the major findings and limitations of this study under discussion and conclusion, respectively.

## Study area and data

**Figure 1:**
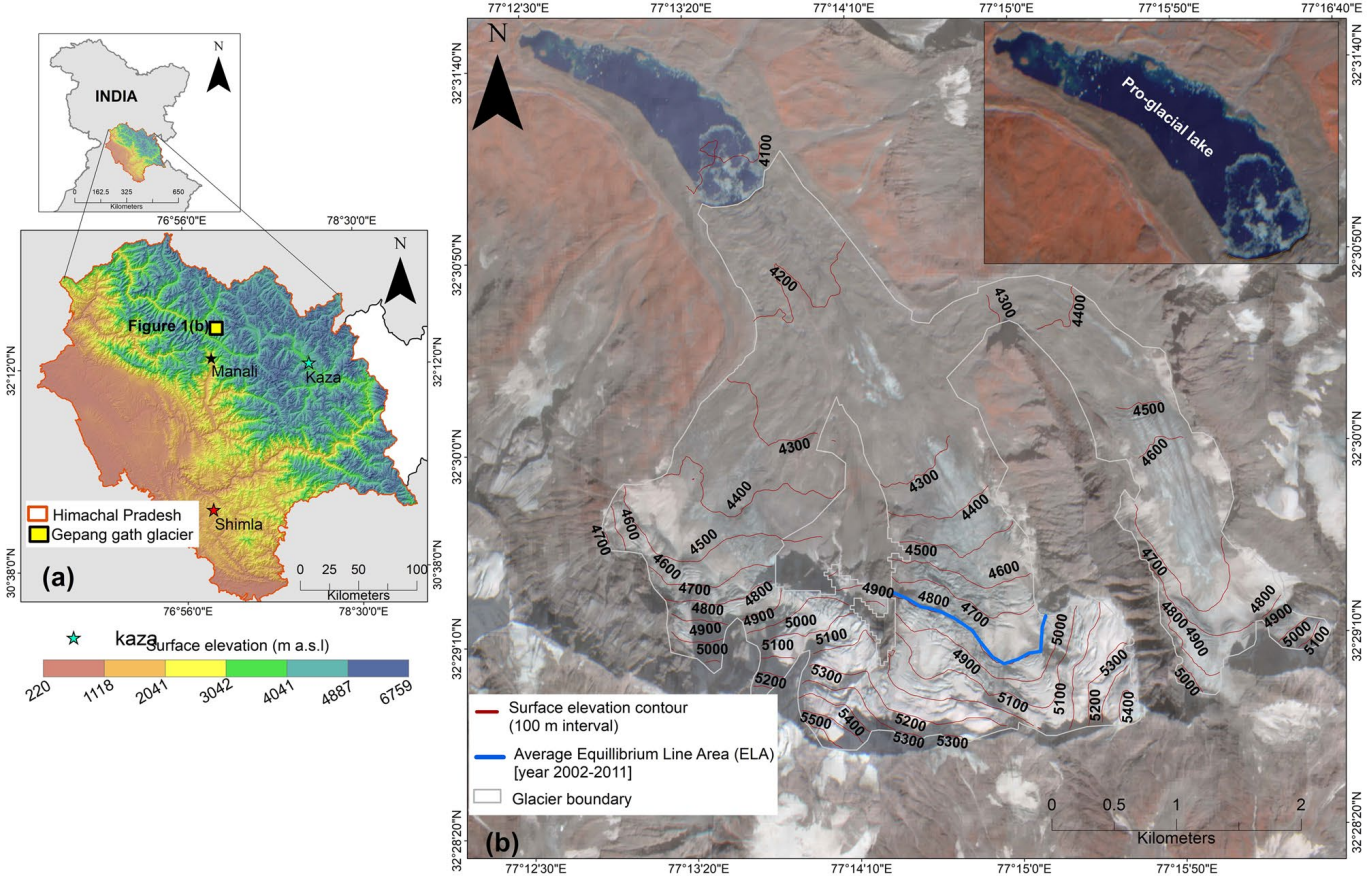
(**a**) The Gepan gath glacier is located in the state of Himachal Pradesh, India. State boundaries are shown in red outlines. The glacier is represented as yellow square. The meteorological stations namely Shimla, Kaza and Manali have been represented by red, green and black coloured stars. The above features have been overlayed over the DEM of that region (downloaded from https://bhuvan-app3.nrsc.gov.in/data/download/index.php). (**b**) The glacier boundary is shown in white outlines and is overlayed on Planet view imagery (dated: 21st October, 2020).the elevation contours (at 100 m intervals) are shown in black coloured lines. A zoomed picture of the Gepan gath lake has been shown on the top right corner. The ELA for the period 2002–2011 has been shown as a blue coloured line. (URL: https://desktop.arcgis.com).

Our study area is the Gepan Gath glacier (Fig. 1). The glacier is located in the Chandra basin in the Western Indian Himalaya. It has an area ~ 14.9 km^2^^15^ and length ~ 6 km. The glacier has three cirques and two tributary glaciers. The glacier feeds into a proglacial lake. The mean elevation of the lake is ~ 4063 m a.s.l. and the lake-basin area is about ~ 0.8 km^2^^17^. During the period 1971–2014, the lake area had increased from 0.1 to 0.6 km^2^ and the lake volume increased by ~ 21.7 million m^3^^15^. Studies conducted by^15^ have shown that apart from atmospheric warming, calving is one the processes that contribute glacier melt into the lake, thereby increasing the lake area.

The debris thickness data was acquired from the global debris thickness maps^18^ gridded over an area equal to 35 m by 35 m. These maps were produced by using the sub-debris melt inversion method^19^ and surface temperature inversion method where the debris thickness is basically expressed as a function of sub-debris melt and debris surface temperature. For more details the reader is requested to refer^18^.

For estimating surface mass balance, daily minimum and maximum temperature records for the period 2002–2012 were acquired from the India Meteorological Department (IMD) observatory at Manali. Manali observatory was chosen because it is the closest station to the study glacier (~ 50 km away; aerially) that had uninterrupted records of maximum and minimum temperatures since 1985. Due to the non-availability of precipitation data from the Manali observatory, the precipitation data from 2002 to 2012 was procured from next closest IMD observatory at Kaza, HP (~ 130 km away; aerially). In addition to that, a constant temperature lapse rate of − 6.5 deg CKm^−1^ was used to altitude-scale the station temperature to the glacier’s location. The same value of temperature lapse rate was also estimated by^20^ using observed station based meteorological data on another intra-basin glacier. The SMB model was run at hourly timesteps. Hourly estimates of mean air temperature were derived using the methodology outlined by^21^ and^22^, where we assume the diurnal variation of temperature to be (a) sinusoidal in nature and, (b) constrained between the daily maximum and minimum temperature values. Surface elevation was extracted from SRTM DEM (30 m spatial resolution), which was captured on February, 2000. In addition to that, we also used TandemX DEM from 2013^14^ for comparing simulated and observed glacier fronts for 2013. This DEM was freely downloaded from https://earthexplorer.usgs.gov/.

The data regarding glacier geometry such as bed elevation, base width and orientation of the sidewalls were derived from the bed topography map of the Gepan Gath glacier for the year 2000. The bed map was acquired from the study conducted by^14^ where they had estimated the bed topography for the years 2000 and 2013 by (a) assuming Shallow Ice Approximation^23^, (b) parameterizing the longitudinal and lateral stresses in the form of a valley shape factor and (c) optimising the shape factor based on glacier surface geometry^24^.

For simulating the past glacier retreat, long term meteorological records are essential^17,21,25^. Therefore, the historical temperature and precipitation records from the IMD observatory at Shimla (~ 250 km away from the glacier; aerially) were acquired for the period 1876–2009 as this was the only observatory with such long records of the mentioned meteorological variables. It shall be noted that records from this meteorological observatory were successfully used by^13^ in modelling the evolution of another glacier that is located in the same river basin as that of the Gepan Gath glacier and hence it is used in this study as well. In this study, information regarding the value of temperature lapse rate was adopted from^13^.

## Methodology

**Figure 2:**
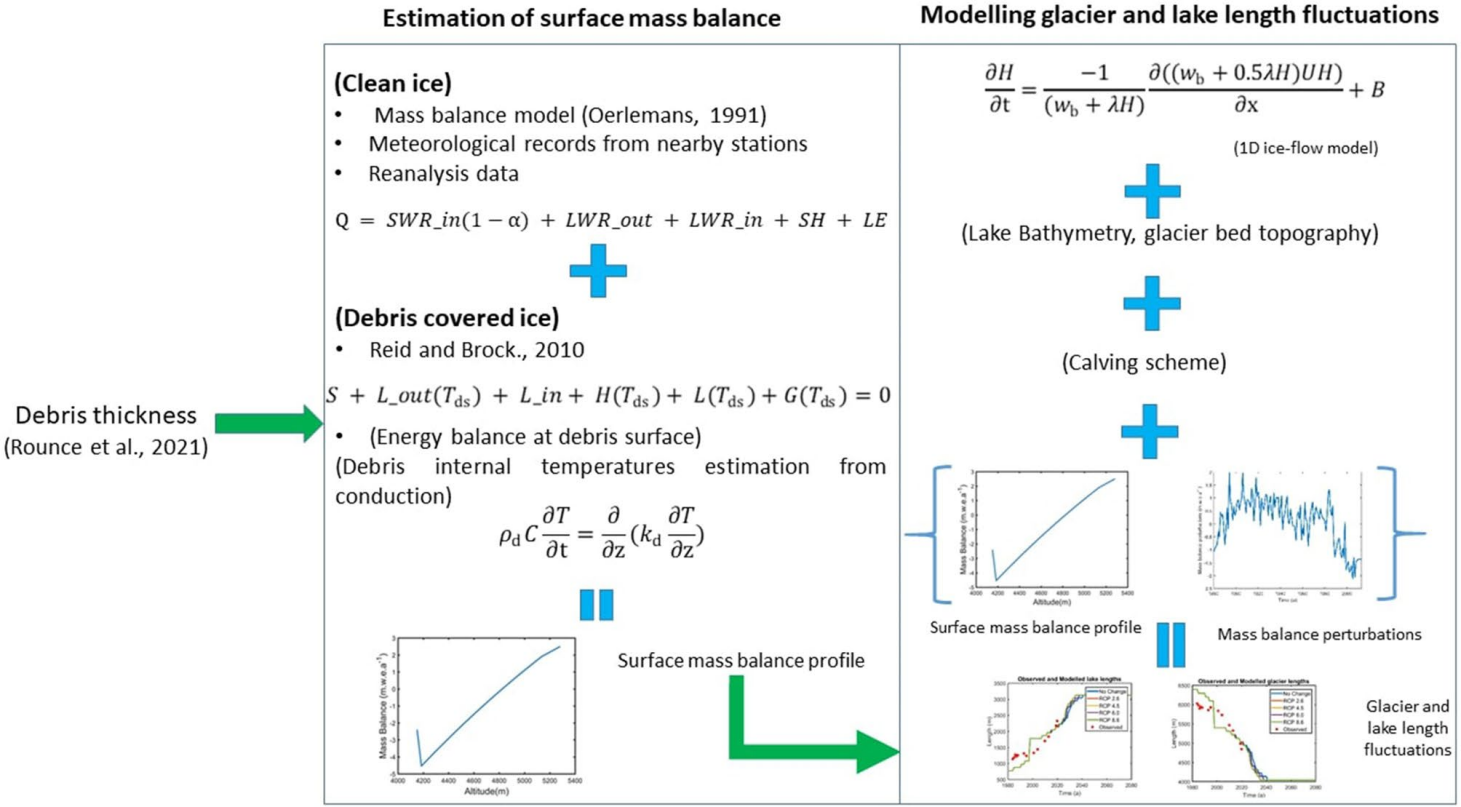
Schematic of the methodology used in this study. Starting from the left, the first block titled ‘Estimation of debris thickness’ briefly summarises how debris thickness was estimated; the second titled ‘Estimation of surface mass balance’ shows the approach used in estimating surface mass balance and finally the last block titled ‘Modelling glacier and lake length fluctuations’ shows the way in which the numerical ice flow model was used for simulating past retreat and past lake length fluctuations. The green arrows between the blocks indicate which variable from one block is used in the analysis of another block. The relevant equations used in each of the blocks have been shown in red colour. (URL: www.mathworks.com).

This section is divided into two major parts (Fig. 2):

### Estimation of surface mass balance

The surface mass balance (SMB) of Gepan Gath glacier is estimated using two different mass balance models, one for the debris free region and the other for debris-covered region.

#### SMB for debris free region

Here, the surface mass balance model proposed by ^22^ is used, where we estimate the surface melt by calculating the surface energy balance. Assuming the glacier surface to be at the pressure melting point and assuming no conduction of heat into the glacier ice and no refreezing, the energy balance on the glacier surface can be expressed as:

1 $${\text{Q }} = { }SWR\_in\left( {1 - {\upalpha }} \right){ } + { }LWR\_out{ } + { }LWR\_in{ } + { }SH{ } + LE{ }$$

where α is surface albedo. *SWR_in* is the incoming shortwave radiation (Wm^−2^). This radiative flux is modelled as the sum of two components namely direct (*Q*_dir_) and diffusive (*Q*_dif_) radiation flux:

2 $$SWR\_in = \tau_{{\text{a}}} \tau_{{\text{n}}} \left( {Q_{{{\text{dif}}}} + Q_{{{\text{dir}}}} } \right)$$

where ${\tau }_{\mathrm{a}}$ and ${\tau }_{\mathrm{n}}$ are transmissivities due to air molecules/aerosols and clouds respectively and are expressed as:

3 $$\tau_{{\text{a}}} = \left( {0.79 + 0.000024h} \right)\left( {1 - 0.08\frac{{\left( {\frac{{\uppi }}{2} - \theta_{{\text{g}}} } \right)}}{{\frac{{\uppi }}{2}}}} \right)$$

4 $$\tau_{{\text{n}}} = 1 - \left( {0.41 - 0.000065h} \right)n - 0.37n^{2}$$

where *h* is surface elevation (m), *ϴ*_g_ is the solar elevation angle. This angle was estimated using standard procedures that are routinely used to estimate solar constant, *n* is cloudiness, *Q*_dir_ and *Q*_dif_ are calculated by using the formulations given in^22^, ${\upalpha }$ is albedo, *LWR_out* is outgoing longwave radiation (Wm^−2^). This flux is modelled by assuming ice surface to be a black body and is modelled as follows:

5 $$LWR\_out = \sigma T_{{{\text{is}}}}^{4} { }$$

where *σ* is Stefan Boltzman constant= 5.67 * 10^−8^ (Wm^−2^K^−4^) and *T*_is_ is ice surface temperature assumed to be 0 °C, *LWR_in* is net incoming longwave radiation (Wm^−2^). This flux is due to the contribution from two sources one from the clear-sky atmosphere and the other from the base of the clouds^22^:

6 $$LWR\_in = e_{{\text{s}}} \sigma T_{{{\text{is}}}}^{4} + I_{{{\text{cl}}}}$$

where *e*_s_ is the emissivity of the lowest tens of meters of the atmosphere layer and is modelled as:

7 $$e_{{\text{s}}} = 0.7 - 0.000025h + 0.000000595e_{{\text{a}}} {\text{e}}^{{\frac{1500}{{T_{{\text{a}}} }}}}$$

where *e*_a_ is the vapour pressure (Pa), *h* is altitude (m) and *T*_a_ is the hourly air temperature (K). Hourly air temperature values are generated from the maximum and minimum temperatures recorded at the weather station (Manali). This is done by assuming (a) a sinusoidal nature of the diurnal variation of air temperature and, (b) a constant lapse rate of − 6.5 Kkm^−1^ (e.g. ^13^). *I*_cl_ is the radiation flux that is emitted from the cloud base and is transmitted in the 8–14 mum band. *SH* is sensible heat flux (Wm^−2^), LE is latent heat flux (Wm^−2^).

*SH* is modelled following^22^:

8 $$SH = 0.0129PAu\left( {T_{{\text{a}}} - T_{{{\text{is}}}} } \right)$$

where *P* is local air pressure, *A* is the von-Karmann constant, *u* is wind speed assumed to be a constant = 2 ms^−1^ (i.e. same as the value of u for Chhota Shigri glacier) and *T*_a_ is air temperature at a height of 2 m from glacier surface.

*LE* is modelled as a function of vapour pressure e_a_:

9 $$LE = 19.8Au\left( {e_{{\text{a}}} - e_{{{\text{as}}}} } \right)$$

where *e*_as_ is the saturated vapour pressure at the ice surface. Finally, the melt *M* (m.w.e. a^−1^) is estimated as follows:

10 $$M = \min \left( {\left( {P_{{\text{r}}} + \frac{Q}{L}} \right),2.5} \right)$$

where *L* is latent heat of fusion of ice and *P*_r_ is the precipitation (m).

Due to the non-availability of cloud base height data, *I*_cl_ (e.q. 6) is neglected. In addition to that, the data on cloudiness (*n*) was also not available for our study area. This data is essential to model the variables mentioned in Eq. 3. Similarly, observed data on the glacier surface albedo was also not available. Albedo is required to solve Eq. (1).

Keeping the above limitations in mind, the following strategy is adopted for modelling surface mass balance:

- Estimation of equilibrium line altitude (ELA): For estimating ELA, the methodology proposed by^26^ was used. Using that method, for every year in the period spanning 2002–2011, Landsat 5 and 7 imagery between June and Oct months were used to delineate the ELA.
- Tuning of cloudiness (*n*) and albedo (*α*): In order to account for the effects of cloudiness and cloud base temperature after step a), *n* was fixed as 0.5 and the value of albedo (*α*) was varied till the modelled mass balance profile had the same ELA as that derived from the satellite imagery.

The complete list of the methodological variables is given in Table S1 in the supplementary text.

The above model was run at hourly timestep and was tested for the Chhota Shigri glacier lying in the same river basin and the results are shown in the supplementary text.

#### SMB for debris covered region

Here, the model proposed by Reid and Brock, 2010 was used. However, instead of using in-situ measured energy fluxes (i.e. as it was done by^27^), we modelled the energy fluxes as explained below. Only a brief description of the model is given here. A detailed model description can be inferred from^27^.

The debris layer is divided into *N* layers each having a thickness of 1 cm. The energy balance at the debris-air interface can be expressed as:

11 $$S{ } + { }L\_out\left( {T_{{{\text{ds}}}} } \right){ } + { }L\_in + { }H\left( {T_{{{\text{ds}}}} } \right) + { }L\left( {T_{{{\text{ds}}}} } \right) + G\left( {T_{{{\text{ds}}}} } \right) = 0$$

where *T*_ds_ is debris surface temperature. *S*, *L_in* represent the incoming shortwave and longwave radiative flux at the debris surface. *L_out*, *H*, *L* and *G* represent the outgoing longwave radiative flux, sensible heat flux, latent heat flux and conductive heat flux respectively. *T*_ds_ is determined by using Newton-Raphson algorithm:

12 $$T_{{{\text{ds}}}} \left( {t + {1}} \right) = T_{{{\text{ds}}}} \left( t \right) - F\left( {{\text{T}}_{{{\text{ds}}}} } \right)/{\text{F}}^{\prime } \left( {T_{{{\text{ds}}}} } \right)$$

where *F′*(T_ds_) is the derivative of *F*(*T*_ds_) with respect to *T*_ds_, is calculated numerically by the central difference method n is the number of iterations. For the first timestep *T*_ds_ is set equal to the air temperature *T*_a_ and thereafter it is set equal to the value of *T*_ds_ calculated for the previous timestep. Eq. (15) is calculated repeatedly until |*T*_ds_(*t*+1)-*T*_ds_(*t*)|<0.01.

After obtaining an estimate of *T*_ds_, the debris internal temperatures are estimated using the heat diffusion equation:

13 $$\rho_{{\text{d}}} C\frac{\partial T}{{\partial {\text{t}}}} = \frac{\partial }{{\partial {\text{z}}}}\left( {k_{{\text{d}}} \frac{\partial T}{{\partial {\text{z}}}}} \right)$$

where ${\rho }_{\mathrm{d}}$ is debris density assumed to be 1496 kgm^−3^, *C* is debris specific heat capacity assumed to be 948 Jkg^−1^ K^−1^ and k_d_ is the debris heat conductivity. At the debris-ice interface, the temperature is assumed to be 273.16 K.

In Eq. (11), *S* is net incoming shortwave radiation (Wm^−2^), *L_in* is the incoming long wave radiation (Wm^−2^)

14 $$L\_in = e_{{\text{s}}} *\sigma *T_{{\text{a}}}^{{4}}$$

where *e*_s_ is the emissivity of the lowest tens of meters of the atmosphere layer and is modelled as:

15 $$e_{{\text{s}}} = 0.{7} - 0.0000{25}h + 0.000000{595}e_{{\text{a}}} {\text{e}}^{{({15}00/T{\text{a}})}}$$

where *e*_a_ is the vapour pressure (Pa), *h* is altitude (m) and *T*_a_ is the hourly air temperature (K). *L_out*(*T*_ds_) is outgoing longwave radiation (Wm^−2^) and is expressed as:

16 $$L\_out = emm\_deb*\sigma *T_{{{\text{ds}}}}^{{4}}$$

where *emm_deb* is debris emissivity and is assumed to be 1. *T*_ds_ is debris surface temperature (K). *H* is sensible heat flux (W/m^2^)

17 $$H = {1}.{29}*{1}0^{{ - {2}}} *P*A*u*\left( {T_{{\text{a}}} - T_{{{\text{ds}}}} } \right)$$

where *P* is local air pressure, *A* is the von-Karmann constant, *u* is wind speed assumed to be a constant = 2 ms^−1^ and *T*_a_ is air temperature at a height of 2 m from glacier surface.*L* is modelled as a function of vapour pressure e_a_:

18 $$L = {19}.{8}*A*u*\left( {e_{{\text{a}}} {-}e_{{{\text{ds}}}} } \right)$$

where *e*_ds_ is the vapour pressure (Pa) at the debris surface. *G* is the conductive heat flux (Wm^−2^) and is expressed as:

19 $$G = k_{{\text{d}}} \left( {T_{{\text{d}}} \left( {1} \right) - T_{{{\text{ds}}}} } \right)(\Delta z)^{{ - {1}}}$$

where k_d_ is the debris surface conductivity and is assumed to be 0.7 (Wk^−1^m^−1^)^28^, *T*_d_(1) is temperature of the debris layer just below the debris surface (K) and *Δz* is the thickness of the debris layer (m). Melt underneath the debris layer (*M*_d_) is calculated from the conductive heat flux at the base of the debris:

20 $$M_{{\text{d}}} = \left( {\left( {k_{{\text{d}}} \left( {T_{{\text{d}}} \left( {{\text{N}} - {1}} \right) - {273}.{16}} \right)\Delta z^{{ - {1}}} } \right)} \right)\left( {\rho_{{\text{i}}} *L_{{\text{f}}} } \right)^{{ - {1}}}$$

where, *N* represents the number of debris calculation layers, *L*_f_ is latent heat of fusion and *ρ*_i_ is the density of ice assumed to be a constant = 900 kgm^−3^.

The complete list of the methodological variables and input parameters is given in Table S1 in the Supplementary text.

### Estimation of glacier retreat

This section is divided into two parts.

Model description: For modelling past and future length fluctuations, the model that is used in this study is based on the principles of mass continuity and Shallow Ice Approximation^17,25^. Assuming a trapezoidal cross-section, x as the direction of glacier flow, the ice flow along the glacier’s central flowline can be expressed as:

21 $$\frac{\partial H}{{\partial {\text{t}}}} = \frac{ - 1}{{\left( {w_{{\text{b}}} + \lambda H} \right)}}\frac{{\partial \left( {\left( {w_{{\text{b}}} + 0.5\lambda H} \right)UH} \right)}}{{\partial {\text{x}}}} + B$$

where, *H* is local ice thickness (m), *w*_b_ is the width of cross-section’s base (m), *λ* parametrises the orientation of the side walls, *B* is the specific mass balance (m w. e. a^−1^) and *U* is vertically averaged mean cross-sectional velocity (ma^−1^) and is modelled as:

22 $$U = f_{{\text{k}}}^{3} \left( {f_{1} \gamma H^{4} \left( {\frac{{\partial h_{{\text{e}}} }}{{\partial {\text{x}}}}} \right)^{2} \left( {\frac{{\partial h_{{\text{e}}} }}{{\partial {\text{x}}}}} \right) + f_{2} \gamma H^{2} \left( {\frac{{\partial h_{{\text{e}}} }}{{\partial {\text{x}}}}} \right)^{2} \left( {\frac{{\partial h_{{\text{e}}} }}{{\partial {\text{x}}}}} \right)} \right)$$

where *γ* = (*ρg*)^3^, *ρ* is ice density and is assumed to have a constant value of 900 kg/m^3^, *g* = 9.8 m s^−2^, *f*_1_ and *f*_2_ represent the deformation and sliding flow parameters and are assumed to be 1.9 × 10^−24^ Pa^−3^ s^−1^ and 5.7 × 10^−20^Pa^−3^ m^2^ s^−1^, respectively^29^, *h*_e_ is surface elevation (m), *f*_k_ is a correction factor that parametrizes the effects of lateral and longitudinal drag^30,31^. In this study, the value of *f*_k_ is assumed to be 1 because lateral and longitudinal stresses do not significantly affect glacier retreat over decadal and multi-decadal time scales^21^. The specific mass balance *B* is further expressed as:

23 $$B\left( {h_{{\text{e}}} ,{\text{t}}} \right) = B_{{{\text{ref}}}} + b\left( {\text{t}} \right)$$

where *B*_ref_ is the reference specific mass-balance profile and is solely a function of *h*_e_. *B*_ref_ is estimated using the surface mass balance model that explained above, *b*(t) represents the balance perturbations derived from proxies such as temperature, precipitation, tree rings etc. *b*(t) is purely a function of time and is applied irrespective of altitude.

### Numerical implementation

Equation (21) is solved numerically along two central flowlines with a horizontal grid spacing of 100 m. A semi-explicit scheme is used for the analyses. In order to accommodate the contribution of ice from cirques and tributary glaciers, the numerical schemes mentioned in^13^ are used.

In addition to that, a numerical scheme had to be developed in order to simulate the movement and mass loss of the glacier snout due to calving. Unfortunately, due to the lack of observed lake bathymetry data, the calving schemes suggested by other previously conducted investigations^18^ did not yield good results in our case. Therefore, in order to account for the mass loss due to the calving mechanism, we adopted a slightly different numerical scheme. This scheme is successful in quantifying the mass loss due to calving but is not particularly successful in simulating the calving front of the glacier’s snout.

In this scheme, we first determined an initial value of the surface elevation of the water level in the lake. This value was tuned till a best possible match between observed and modelled lake lengths are obtained. This initial surface elevation was assumed to be 4050 m, which is also the observed value in 2017 as reported by^15^ (personal communication). The tuning was done in the following way:

At every time-step, the position of the terminus is shifted to the point where the surface elevation of the glacier front was equal to 4050—H_e_ m. Where H_e_ is assumed to be less than or equal to 1% of the water level. As a result, the terminus may move forward, backward or stand still owing to being in a state of advance, retreat or equilibrium respectively. The actual position of the terminus is then computed by interpolating between values of two neighbouring grid points with surface elevation larger and smaller than 4050—H_e_ m. Thereafter, new grid points are defined to fit the updated glacier length, and the surface elevation is recalculated for the new grid points by using piecewise cubic interpolation. Ideally, the value of H_e_ should vary with time due to an increase in the lake level owing to the accumulation of glacier melt water but for the sake of simplicity, it was decided to use a constant value of H_e_ throughout the model simulation. In our analyses, a good agreement between the observed and modelled lake length fluctuations is obtained with H_e_ equal to 40 m. As a result of the above interpolation, the spatial grid resolution varied between 90 and 100 m.

Using the aforementioned methodology, we simulated the advance of the glacier from no ice to its maximum length followed by a retreat. The model assumes that the depression was excavated due to glacier motion and that there is no calving during the advance stage as the depression was probably filled with unlithified sediments^9^. Consequently, the calving module was activated during the glacier retreat.

## Results

This section has been divided into three sub-sections. In the first part, we discuss the modelled surface mass balance profile of Gepan Gath glacier, in the next section we discuss the simulation of glacier retreat over the past 134 years and finally in the last sub-section we discuss the evolution of the glacier-fed proglacial lake.

### Modelled mass balance of the glacier

**Figure 3:**
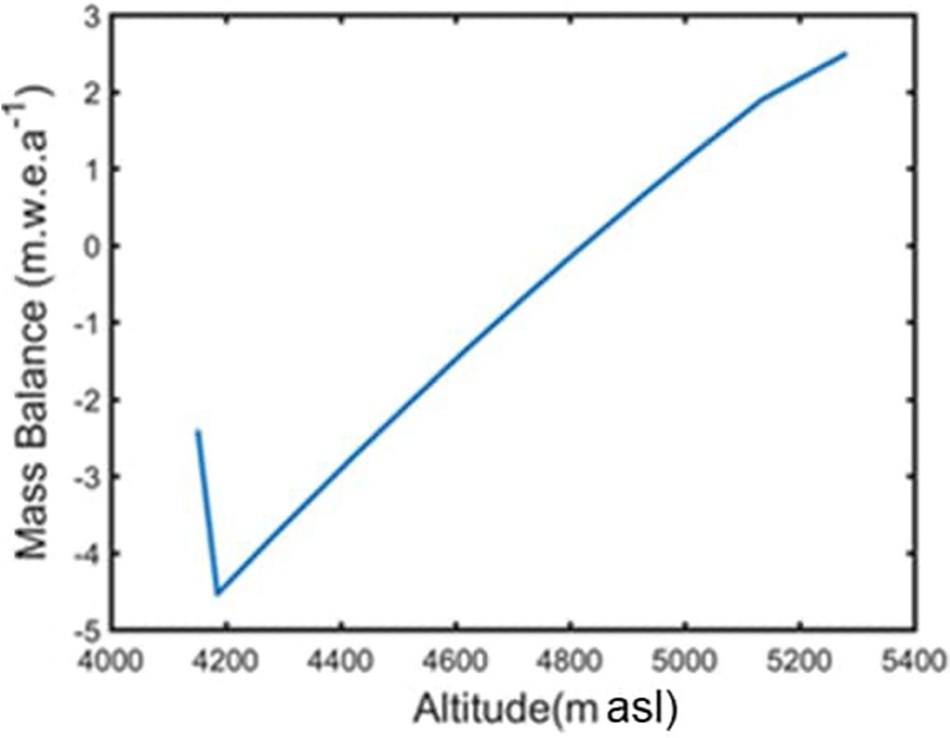
Mean, annual mass balance profile of the main stream of the Gepangath glacier over the period 2002–2011. This profile was modelled by using the ERA reanalysis data and daily meteorological records from the IMD observatories at Manali and Kaza. The mass balance profile for the tributary was estimated by adjusting the main stream’s mass balance curve wrt altitude of the tributary. (URL: www.mathworks.com).

The mean mass balance profile of the main trunk and the tributary of Gepan Gath glacier for the period 2002–2011, is shown in Fig. 3. The variation in the ELA of the glacier has been given in Table S2 in the Supplementary text. For the main trunk, the mean mass balance profile decreases from ~ 2.5 m w. e. a^−1^ at ~ 5000 m altitude to ~ − 5 m w. e. a^−1^ at ~ 4200 m. From 4200 m onwards, i.e. in the heavily debris covered region, the mean mass balance increases from ~ − 5 m w. e. a^−1^ to ~ − 2.3 m w. e. a^−1^ which is in line with the general fact that thicker supraglacial debris cover insulates the glacier surface to some extent thereby increasing the mass balance. The mass balance curve for the tributary glacier is derived by correcting the main stream’s balance curve for altitude. Due to the non-availability of observed mass balance data for the Gepan Gath glacier, the mass balance model was validated by comparing the modelled glacier-wide annual mass balance for the Gepan Gath glacier with the remote sensing-based estimates reported by^32^. The comparison is shown in Table 1, which indicates that the present study estimates and that by^32^ differ by ~ 0.31 m w. e. a^−1^. Likewise, our estimates of glacier wide mass balance were compared with the bi-decadal estimates reported by other independently conducted, published investigations. The published estimates were estimated by using geodetic technique^18^. As an example, for the period, 2000 to 2018 the average annual glacier-wide mass balance of Gepan Gath glacier estimated in this study and that in^18^ was − 1.2 m w. e. a^−1^ and − 0.86 ± 0.361 m w. e. a^−1^, respectively. Differences between these estimates are not high. Thus, one can say that our modelled glacier wide mass balance estimates compared well with other published ones. In addition to that, the mass balance model was validated on the nearby debris covered glacier namely the Chhota Shigri glacier where observed records of the altitudinal profile of surface mass balance was available for the period 2002–2011^20^. The result is shown in the Online Appendix (Figure A1). The model seems to well-replicate the observations in the case of Chhota Shigri glacier. Given the good match between observed and modelled values, we believe that our estimates of surface mass balance for the Gepan Gath glacier is reliable.

**Table 1:** Comparison between the glacier mass balance estimates obtained in this study and that obtained by Sayali et al., 2017.

Year	Mass balance estimates from Current work (m.w.e. /a)	Mass balance estimates from Sayali et al. 2017 (m.w.e. /a)
2002	− 1.26	− 1.22
2003	− 1.45	− 1.17
2004	− 1.11	− 1.22
2005	− 0.84	− 0.77
2006	− 1.53	− 1.17
2007	− 1.67	− 1.23
2008	− 1.04	− 1.23
2009	− 1.27	− 0.81
2010	− 1.17	− 1.01
2011	− 0.53	− 1.09

### Modelled past retreat of the glacier

**Figure 4:**
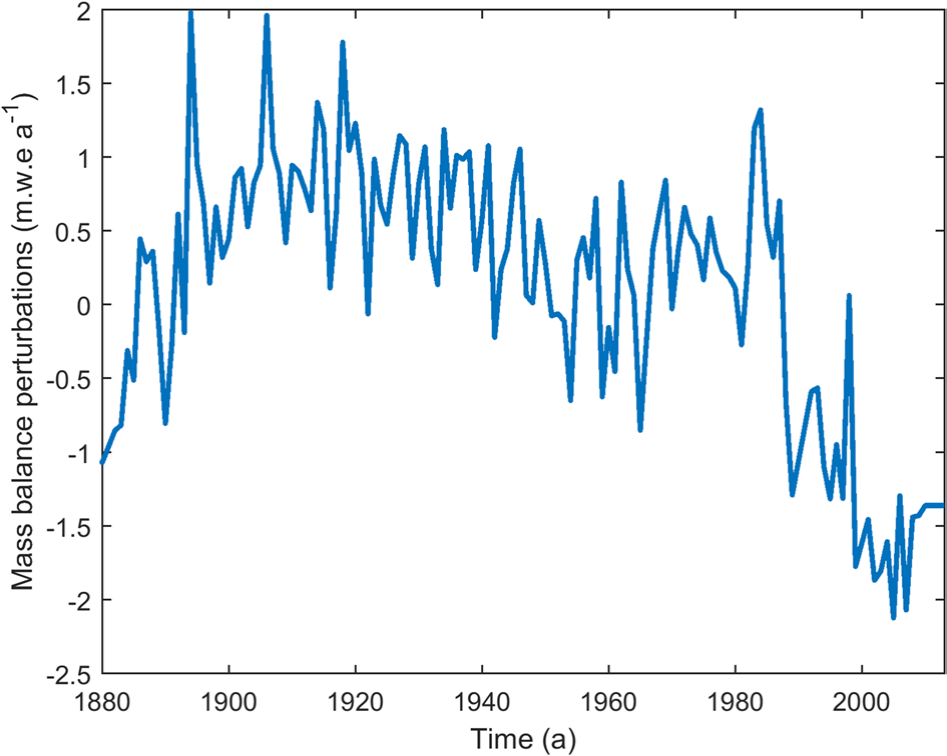
The annual mass balance perturbations are yearly averages of the respective annual mean temperature annomalies. The perturbations were used to simulate the length fluctuations. (URL: www.mathworks.com).

For simulating the historic retreat of the past 134 years, the ice-flow model was forced with the mass balance perturbations (*b*(t)) shown in Fig. 4. These values were derived from the temperature data derived from the IMD station at Shimla^14^. ^14^ reported that temperature was the major meteorological variable that influenced glacier retreat on decadal, multi decadal timescales. Due to the close proximity of the Gepan Gath glacier to the study area of^13^, we assumed that *b*(t) is solely a function of air temperature anomalies. Mass balance perturbations was estimated by assuming a linear relationship between the mass balance and temperature anomalies as explained in^13^.

**Figure 5:**
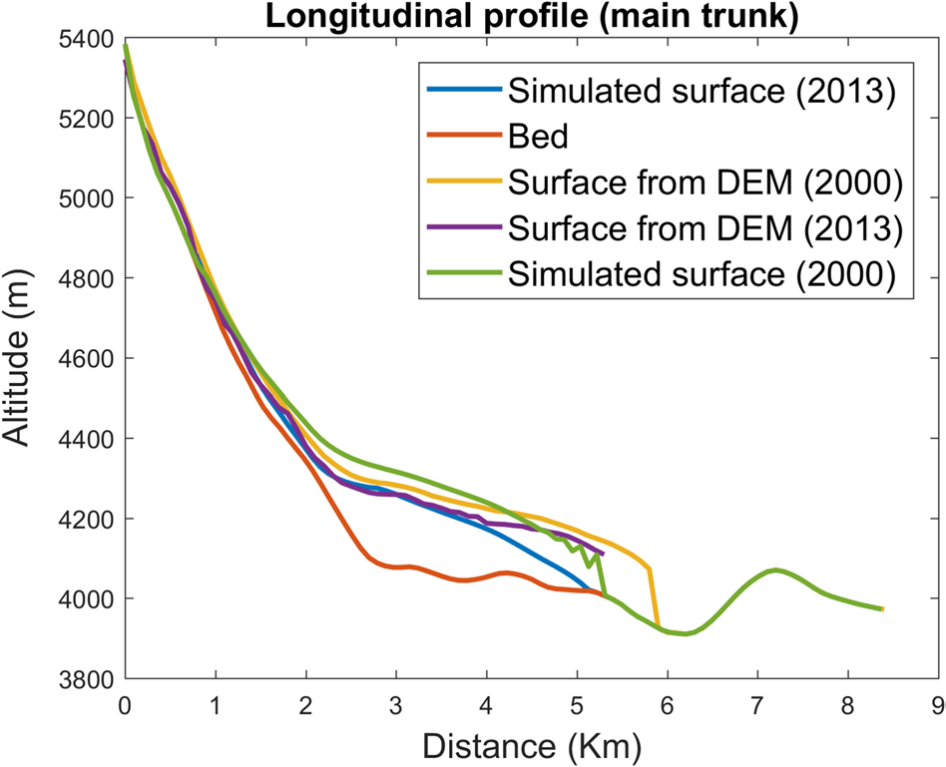
Shows the simulated and observed glacier surfaces for the year 2000, 2013 for the mainstream. (URL: www.mathworks.com).

**Figure 6:**
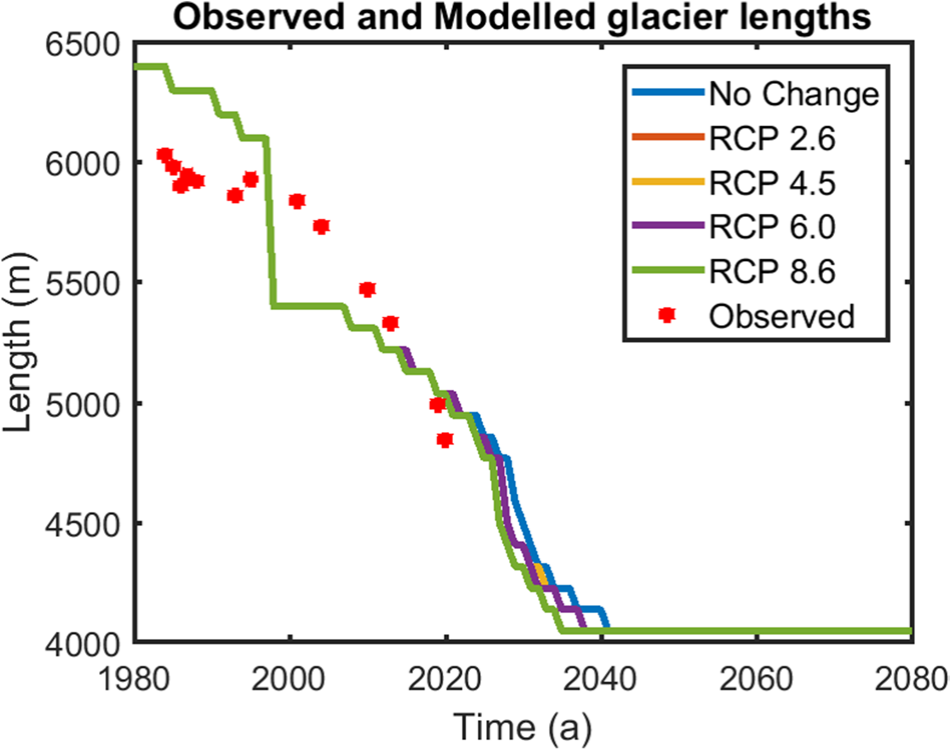
Future glacier length fluctuations of the Gepangath glacier under RCP 2.6, RCP 4.5, RCP 6.0 and RCP 8.6 scenarios. (URL: www.mathworks.com).

A comparison between the simulated and observed glacier fronts from SRTM DEM (2000) and the TanDEM-X DEM (2013) is shown in Fig. 5. The simulated glacier front was behind the observed glacier front by 400 m during 2000. However, in the year 2013, the simulated and Tandem-X DEM^14^ derived glacier front were barely 200 m apart. A comparison of the simulated and observed glacier length fluctuations is shown in Fig. 6. Contrary to glacier surface elevation, particularly good agreement was observed between the simulated and Google-Earth images based derived glacier length fluctuations for the period 1984–2020, wherein the RSME was ~ 320 m. The maximum difference obtained was ~ 440 m and the minimum difference was ~ 110 m.

### Modelled glacial lake lengths

**Figure 7:**
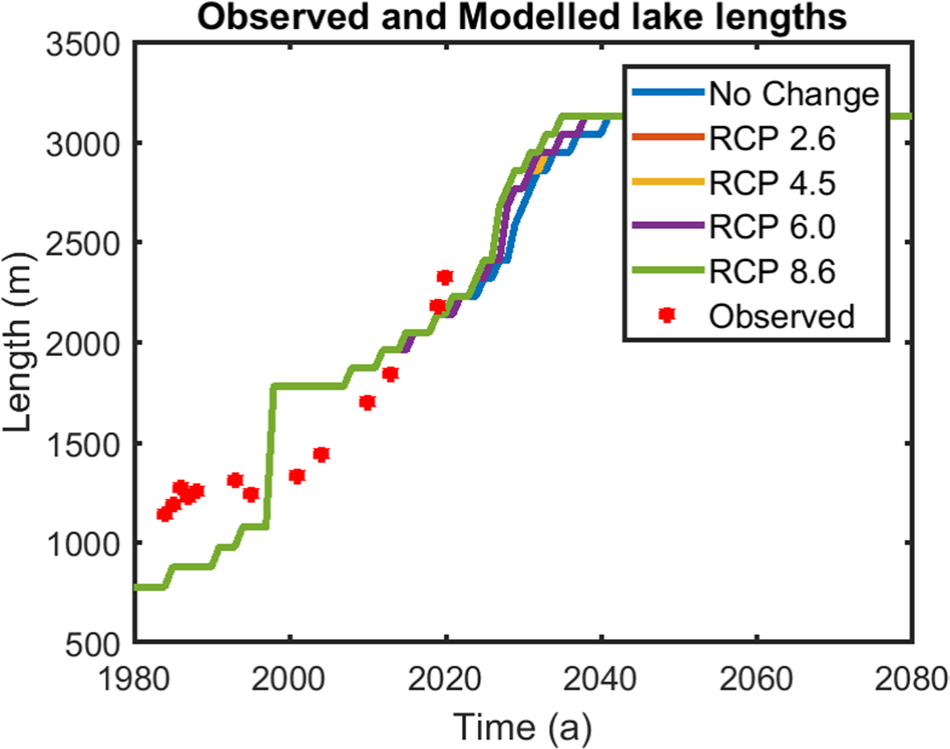
Lake length fluctuations RMSE between observed and modelled lake lengths ~ 110 m with maximum and minimum deviation of 173 m and 20 m respectively. (URL: www.mathworks.com).

In addition to the above, the modelled and Google Earth images based manually derived lengths of the Gepan Gath lake along the central flowline at different times show good agreement with each other (Fig. 7, Table 2). In 1984, observed length was ~ 1146 m and the modelled lake length was ~ 880 m. In 1993, the Google-earth-image based observed and the modelled lake lengths were 1316 m and 1080 m respectively. In the next 11 years we see that the difference between modelled and the observed lengths narrowing down with observed and modelled lake lengths in 2020 being 2329 and 2141 m respectively. We also compared our modelled lake lengths with that estimated using remote sensing techniques on 5-year mosaics of satellite imagery^3^. For the period 2004 to 2008, the lake length estimated by our modelling was 1445 m compared to 1491 m by^3^ estimates. The difference was 3%. Similarly, for the period 2014 to 2018 our modelled estimates (1846 m) and the estimates (2192 m) by^3^ differed by ~ 15% only. From the above comparisons, we safely conclude that our modelled results are reliable.

**Table 2:** Comparison between the modelled and observed lake lengths.

Year	Modelled (m)	Observed (m)
1984	880	1146
1993	1080	1316
2004	1781	1445
2013	1961	1846
2019	2141	2183
2020	2141	2329

## Uncertainty analyses

**Figure 8:**
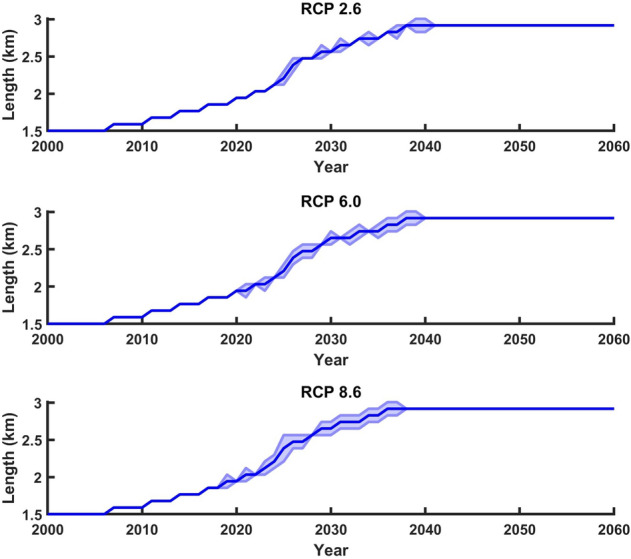
Uncertainty associated with the future lake lengths. The shaded regions represent the uncertainty in the lake lengths. (URL: www.mathworks.com).

In order to calculate uncertainty in the estimated future lengths of the Gepan Gath lake, the maximum and minimum temperature anomalies in 2100 and 2011 for the respective RCP scenarios were estimated. For this purpose, we referred to the CMIP5 temperature projections in Himalaya-Karakoram which is shown in^33^. For RCP 2.6 scenario, the maximum and minimum temperature anomaly in AD 2100 were 3 °C and 0 °C^33^. In the case of RCP 6.0, the maximum and minimum temperature anomalies for the year AD 2100 were 7 °C and 3 °C respectively^33^. Similarly, for RCP 8.6 the maximum and minimum temperature anomalies for the year AD 2100 were 4.4 °C and 9.8 °C respectively^33^. The maximum and minimum temperature anomalies for the year 2011 were 0.16 °C and 1.5 °C respectively. In every RCP scenario, we estimated the slopes of the curves joining the minimum and maximum temperature anomalies in 2011 to their corresponding counterparts in AD 2100. Once the slopes were estimated, we applied Eq. 26 to derive the corresponding mass balance changes between 2011 and AD 2100. These mass balance perturbations were used to force the numerical ice flow model to estimate the corresponding fluctuations in the lake lengths (Fig. 8). The thick blue line in each of the plots represent the lake length fluctuations, which is shown in Fig. 7. The shaded regions represent the uncertainty in the lake length fluctuations. The maximum and minimum uncertainty are ~ 200 m and ~ 100 m respectively.

## Discussion

The first available satellite imagery derived sighting of the Gepan Gath lake was in 1971^15^. In that year, the observed lake surface area was ~ 0.1 km^2^. Our modelling suggests that in the same year the lake surface area was ~ 0.2 km^2^. This difference is probably the result of the idealistic assumption of trapezoidal cross-section of the glacier. In the same year, the lake length estimated by our model is 200 m.

After having simulated the lake’s historic evolution, four different Representative Concentration Pathway (RCP) climatic scenarios were analysed for predicting the lake’s future evolution. The scenarios were suggested in the AR5 section of the 2014 IPCC report. These scenarios were RCP 2.6, RCP 4.5, RCP 6.0 and RCP 8.6. These scenarios predict a temperature rise of 2.36, 3.49, 3.68 and 5.51 K respectively with respect to 1860–1990 mean, in the Karakoram and Western Himalaya over the next 100 years under a constant precipitation scenario^33^. Figure 6 shows the glacier length variations from 2009 until 2100 under different RCP scenarios. The glacier is expected to retreat by 1.6 and 1.7 km for 2.36 and 3.49 K rise in the mean temperature respectively (i.e. under RCP 2.6 and 4.5 scenarios). The results for RCP 6.0 scenario (3.68 K rise) were found to be similar to that of RCP 4.5. The glacier is expected to lose > 90% of its volume before 2040 for a 5.51 K rise in mean temperature i.e. under RCP 8.6 scenario. The nature of the depression in the glacier bed topography as derived from^14^ shows that with respect to the lake length in 2013, the Gepan Gath lake can grow by at least 420 m along the central flowline in the future. Patel et al.^15^ reported the areal extent of the lake was ~ 0.8 km^2^ in 2014. In comparison to that, our modelled lake area in 2014 is ~ 1.3 km^2^. Under RCP 2.6 and RCP 4.5 scenarios, the lake is expected to grow in length by ~ 420 m by 2030. Similarly, under RCP 6.0 scenario, the maximum length extent will be reached by 2028. Finally, under RCP 8.6 scenario i.e. for a 5.5 K increase in the mean temperature, the glacier fed lake will grow to its maximum length by 2027.

The reference mass balance profile (*B*_ref_) and the glacier bed-rock topography are important inputs in this modelling approach. Due to non-availability of observed surface mass balance estimates, *B*_ref_ was derived using daily meteorological data such as station recorded air temperature and precipitation. Due to non-availability of station recorded data on relative humidity and wind speed, ERA-5 reanalysis derived data for the respective variables were used in our analyses. Records of station temperature and precipitation were publicly available only till 2012. Therefore, the estimated *B*_ref_ was averaged over the period 2002–2012. Similarly, due to the unavailability of in-situ bed elevation data for the Gepan Gath glacier unlike other past studies such as^9^, the bed topography given in^24^ was used.

**Figure 9:**
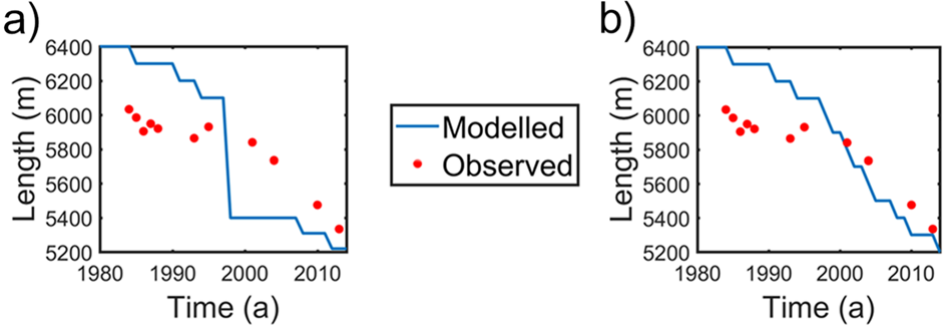
A comparison of glacier length fluctuations with and without calving scheme. (**a**) Calving. (**b**) No Calving. (URL: www.mathworks.com).

In order to understand the relative role of the glacier-fed lake in influencing the glacier retreat, we compared the length fluctuations derived between a calving scenario (Fig. 9a) and a no-calving scenario (Fig. 9b). In the absence of a calving mechanism, in the period 1990–2000, the modelled length fluctuations are smooth and non-abrupt as compared to that modelled using the calving mechanism suggesting that calving enhances mass loss (Fig. 9b). We believe that this abrupt change in glacier length is a combined effect of the uncertainties in *B*_ref_ and bed topography as explained earlier. It is likely that due to these uncertainties, the model is unable to simulate the glacier front accurately. However, in spite of the deficiencies, the model gives reasonably good estimates of overall length fluctuations of the lake, which is what this study aims at simulating. This study shows that even with limited data availability, the glacier-fed lake evolution can be modelled with at least moderate success.

Keeping the obtained results in mind, we believe that our study provides a methodology to help in monitoring the evolution of glacier-fed lakes that are in critical state, in extremely data-sparse regions such as the Indian Himalaya. Numerical ice-flow modelling based studies have the ability to reasonably predict the growth or decay of the glacier lakes.

The bed topography also plays an important role during the retreat/ advance process. Past studies have shown that the local slope of glacier bed is a detrimental factor in deciding the rate or advance/retreat^25^. We believe that the model results can be further improved if observed or Ground Penetrating Radar (GPR) derived bed elevation, in-situ lake bathymetry data is used instead of a model derived bed elevation. Using observed bed elevation might reduce the topography related uncertainties in the glacier length and subsequently the lake length variations respectively.

## Limitations

Though we were able to simulate the past as well as future lake length fluctuations reasonably well, we still feel that such investigations can be greatly improved if we have observational records on the following things:

- Lake bathymetry: This is a crucial parameter that determines the rate of glacier retreat and the glacier-fed lake’s subsequent expansion. The deeper and larger the lake, the more pronounced will be the glacier’s mass loss due to calving and consequently, the more rapid will the lake’s expansion be. In the case of Gepan Gath glacier, due to the highly treacherous terrain of the glacier and also of the areas surrounding the lake, we were unable to carry out any SONAR sounding investigations of the lake. However, due to non-availability of published data on lake bathymetry, we had to rely on the bed elevation map of Gepan Gath glacier produced by^14^.
- Elevation of the calving front: The calving scheme used in the study is based on the height-above-buoyancy or floatation model as proposed by^34^. This model assumes that the position of the calving front is controlled by the fraction of glacier frontal ice thickness that lies above the lake’s water level. Due to the non-availability of such data and our inability to measure this parameter in the field owing to the extremely rough terrain at the glacier front, we had no choice but to modify the numerical scheme for simulating calving. Consequently, the difference between observed and modelled glacier and lake lengths increased.
- Surface mass balance of the glacier: Due to the non-availability of published record of SMB profile for the Gepan Gath glacier, we were forced to rely on meteorological records from relatively far located weather stations and reanalysis data. As a result, this also added uncertainty to the modelled results. More importantly, due to the non-availability of observed data on SMB, we were forced to calibrate some of the SMB parameters on Chhota Shigri glacier.

These three limitations can be found at most of the glaciers located in the IHR. In spite of that, our analyses show that we can still simulate glacier and glacier-fed lake evolution with reasonable accuracy. We therefore recommend more number of field-based investigations regarding measuring lake bathymetry. We also recommend installation of more weather stations for long term monitoring so that the SMB modelling can be made more accurate than what it is now.

## Conclusion

In this study we modelled the past and future evolution of the Gepan Gath glacier and its moraine dammed lake. This study is a first of its kind that has been conducted in the Indian Himalaya where reanalysis, station data and satellite imagery were used to model the evolution of a large glacier-fed lake. Observed and modelled lake length fluctuations matched reasonably well with each other. Analyses showed that the modelled lake length increased from 880 m in 1984 to 2141 m in 2020. The mean RMSE between the observed and modelled lake lengths is ~ 110 m. In addition to that, we also estimate the evolution of the glacier as well as the glacier-fed lake under different climatic scenarios. Model results suggest that for a 5.5 K rise in the mean temperature, the Gepan Gath glacier is expected to lose more than 90% of its 2010 volume by 2040. Regarding evolution of the lake, our analyses show that, the lake would grow by 420 m at the maximum in the future as predicted by earlier studies. This maximum length will be reached by 2030 under all the climatic scenarios. This study demonstrates the usefulness of a combination of numerical models (such as 1D ice flow and mass balance models) along with satellite imagery for modelling the evolution of critical glacier-fed lakes in data sparse regions such as the Indian Himalayan Region (IHR). We believe that such investigations would be very helpful in monitoring and predicting the evolution of other critical lakes located in the Himalaya and other such data spare regions.

## Supplementary Information

Supplementary Information.

## Data Availability

1. Daily mean temperature and precipitation data were procured from the India Meteorological Department (IMD). This data is freely available from the IMD but cannot be shared by us. The reviewers and readers must request the IMD for access to the data. 2. For estimating debris thickness, Landsat 7 imagery from https://earthexplorer.usgs.gov/ was used. These images can be freely downloaded from the above stated link. 3. Data regarding wind speed, relative humidity were taken from ECMWF reanalysis data, which is freely available from https://www.ecmwf.int/en/forecasts/datasets/reanalysis-datasets/era5. 4. Mass balance data for Chhota Shigri glacier was published in^6^ and data procurement was done from http://www.glims.org/maps/gtng. 5. The data regarding RCP scenarios was published in^11^. 6. The model code can be made available on request. Kindly drop the request at p.gantayat@lancaster.ac.uk.
