# Supplementary material for: Modelling evolution of a large, glacier-fed lake in the Western Indian Himalaya
Source: Sci Rep. 2023 Feb 1;13:1840. doi: 10.1038/s41598-023-28144-8 (PMC9892038; doi:10.1038/s41598-023-28144-8)
Supplement: Supplementary file 1 — Supplementary Information. [file 41598_2023_28144_MOESM1_ESM.pdf]

Contents of this file

Figure A1

This supporting information provides the figure discussed in the main article

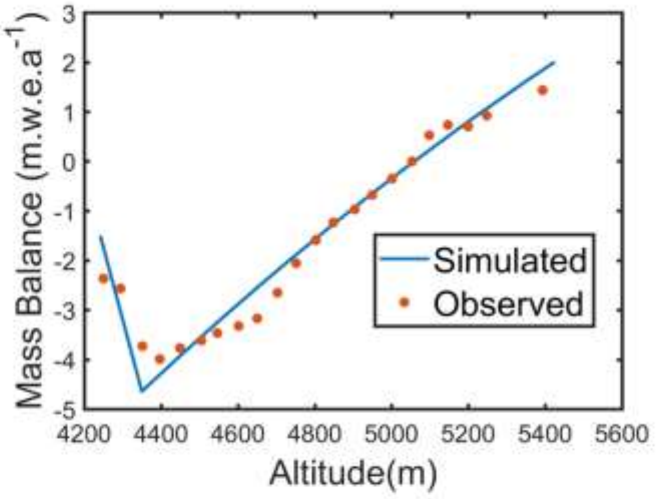

Figure A1: Shows the surface mass balance profile of the Chhota Shigri glacier for the period 2002-2011. Red dots indicate observed mass balance values. Blue represents modelled mass balance. This was used to test the validity of the mass balance model.

Table S1: Variables and parameters used in the mass balance calculations

Variables / Parameters	Name	Value
Θ_g	Solar angle	
σ	Stefan Boltzman constant	$5.67 * 10^{-8}$ ($Wm^{-2}K^{-4}$)
A	Von Karmann constant	0.41
u	Wind speed	2 ms^{-1} (from reanalysis data)
L	Latent heat of fusion	$3.34*10^5$ $kJkg$-$^1K^{-1}$
n	Mean cloud cover	0.4
k_d	Debris conductivity	0.7 $Wm^{-2}K^{-1}$ (From published data of other Himalayan glaciers)
α_d	Debris albedo	0.3 (From published data of other Himalayan glaciers)
ρ_d	Debris density	1496 kgm^{-3}
Δz	Thickness of debris layer division	0.01 m
ρ_i	Ice density	900 kgm^{-3}

emm_deb	Debris surface emmissivity	1 (Reid and Brock, 2010)
C	Debris specific heat capacity	948 $Jkg^{-1}K^{-1}$
N	Number of debris layers	40
	Temperature Lapse rate	-6.5 Kkm^{-1}
Δt	Timestep for mass balance model	1 hour

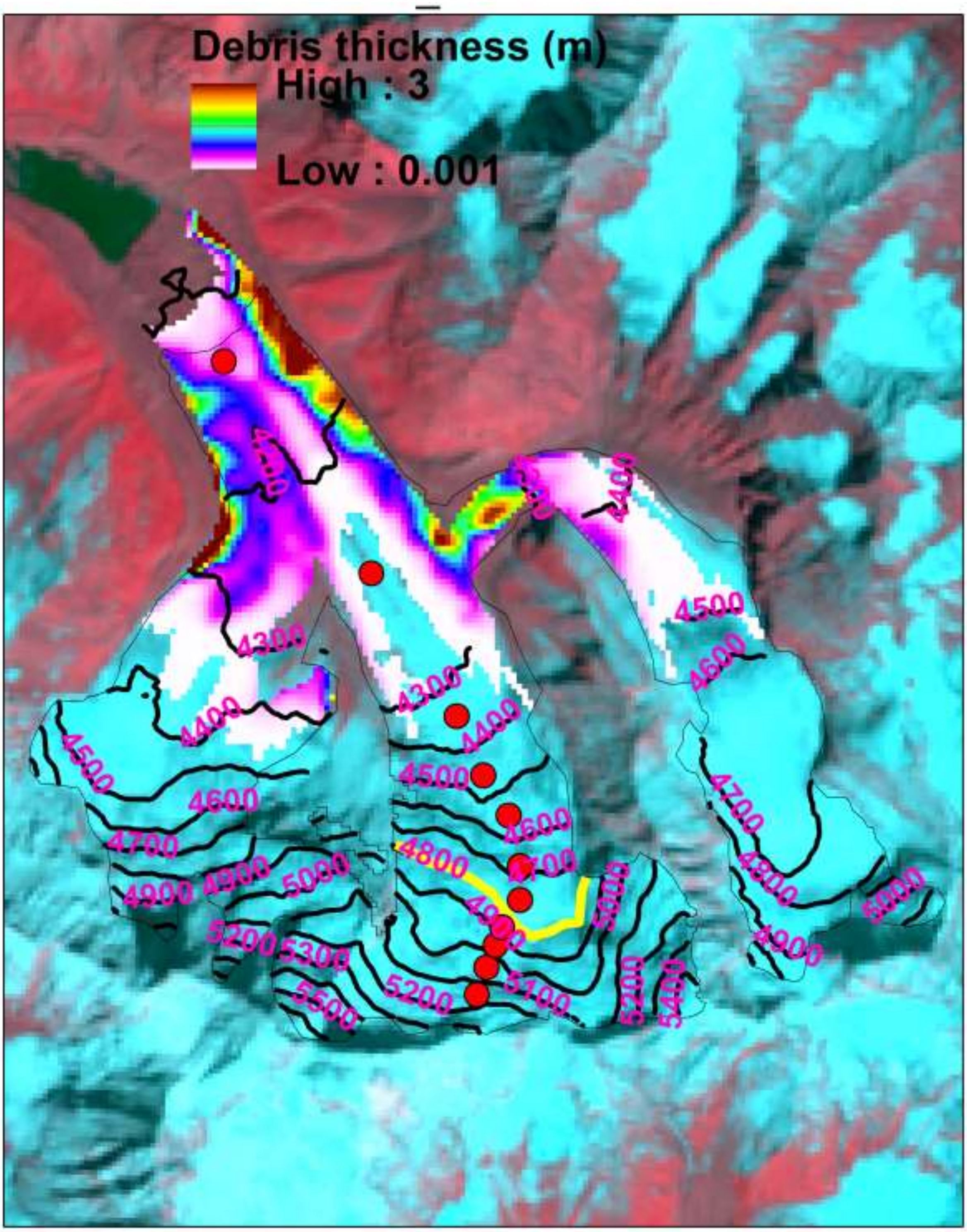

Figure A2: Shows the debris thickness of a region of Gepan gath glacier that was considered for estimating the surface mass balance profile. The background image is a Landsat ETM+ imagery for the year 2002. The black coloured lines represent the surface elevation contours at 100 m intervals. The red coloured dots are the points

where the mass balance was estimated for the period 2002-2011. The ELA for the period 2002 till 2011 is marked as a yellow line. The figure was created using ArcMap 10.1. (URL: https:// desktop.arcgis.com)

Table S2: ELA variation for the period 2002-2011

Year	ELA
2002	4806 m
2003	4806 m
2004	4806 m
2005	4622 m
2006	4806 m
2007	4806 m
2008	4806 m
2009	4806 m
2010	4806 m
2011	4622 m
